# Extracorporeal shockwave therapy: A systematic review of its use in fracture management

**DOI:** 10.4103/0019-5413.50851

**Published:** 2009

**Authors:** BA Petrisor, Selene Lisson, Sheila Sprague

**Affiliations:** 1 Division of Orthopaedic Surgery, Department of Surgery, McMaster University, Ontario, Canada; 2 Department of Clinical Epidemiology and Biostatistics, McMaster University, Ontario, Canada

**Keywords:** Extracorporeal shockwave therapy, delayed union, nonunion, fresh fracture

## Abstract

Extracorporeal shockwave therapy is increasingly used as an adjuvant therapy in the management of nonunions, delayed unions and more recently fresh fractures. This is in an effort to increase union rates or obtain unions when fractures have proven recalcitrant to healing. In this report we have systematically reviewed the English language literature to attempt to determine the potential clinical efficacy of extracorporeal shockwave therapy in fracture management. Of 32 potentially eligible studies identified, 10 were included that assessed the extracorporeal shockwave therapy use for healing nonunions or delayed unions, and one trial was included that assessed its use for acute high-energy fractures. From the included, studies' overall union rates were in favor of extracorporeal shockwave therapy (72% union rate overall for nonunions or delayed unions, and a 46% relative risk reduction in nonunions when it is used for acute high-energy fractures). However, the methodologic quality of included studies was weak and any clinical inferences made from these data should be interpreted with caution. Further research in this area in the form of a large-scale randomized trial is necessary to better answer the question of the effectiveness of extracorporeal shockwave therapy on union rates for both nonunions and acute fractures.

## INTRODUCTION

In the management of fractures, nonunions and delayed unions continue to be significant complications.^1^–^3^ They can result from a confluence of patient factors such as smoking, diabetes, vascular disease or other comorbidities, or injury factors such as high-energy trauma or significant soft tissue loss.^4^–^6^ Nonunions or delayed unions may then result in further surgery with subsequent prolonged or repeat hospitalization, disability, and delays in returning to the workforce.^1^,^7^ The costs associated with these are not insignificant and they can include both personal and societal costs such as lost wages and productivity as well as direct health care costs.^2^ Alternative, less expensive nonsurgical methods of managing nonunions and delayed unions could potentially lessen the impact felt from these entities from both a patient and economic perspective.

Initially used for the treatment of urinary, kidney, and salivary stones, extracorporeal shock wave therapy has been used more and more as a noninvasive treatment modality for nonunions and delayed unions.^8^–^10^ Indeed, initial basic science work using dog and rabbit nonunion models assessed the efficacy of extracorporeal shock wave therapy on obtaining union. This work has suggested that shockwave therapy promotes callous formation as well as a dose-dependent osteogenesis.^11^–^16^ Furthermore, the callous produced appears to undergo appropriate remodeling to lamellar bone. More recently, the bone treated with shockwave therapy has been shown to be associated with neovascularisation and an increased expression of angiogenic growth factors suggesting that increased vascularity may play a role in osteogenesis.^15^ Indeed Maier *et al*. in a rabbit femora model found altered blood flow to the bone treated with shockwave therapy in a dose-dependent fashion.^16^ Further molecular data suggest that there is a direct stimulant effect of shockwave therapy on the differentiation as well as proliferation of cultured osteoblasts.^17^ Also, regulation of genes involved in osteoblast proliferation and differentiation (such as BMP-inducible kinase and prostaglandin E2 receptor for example) has been observed following treatment with extracorporeal shockwaves.^17^

Mechanistically, the shockwave is first generated in water and from there it is transferred through a medium to the skin and tissues as a sonic pulse. This creates expansion and compression within the bone.^18^ In order to be the most beneficial, the pulses must be concentrated on the point of treatment, in this case the nonunion or fracture.^18^ The two basic effects of the shockwave on tissue are direct and indirect.^18^ That is, shockwaves generate mechanical tensile forces within the bone that in turn results in cavitation forces.^18^ These effects have been seen to cause hematoma formation, cell death, and subsequent new bone formation.^18^–^20^

There have been several clinical observational studies demonstrating the effects of shock wave therapy on the healing of bones.^8^,^9^,^20^,^22^ This systematic review attempts to summarize the current clinical literature published on shockwave therapy and more specifically its effects on union rates in relation to acute fracture and nonunion management.

## MATERIALS AND METHODS

### Search strategy

We identified peer-reviewed relevant studies using a systematic search of PubMed, EMBASE, and the Cochrane Collaboration database up to December 31, 2008. The electronic search was tailored to each database in order to locate articles that met the eligibility criteria as described below. We also searched the online meeting archives of the American Academy of Orthopaedic Surgeons and the Orthopaedic Trauma Association from their inception to December 31, 2008. In addition, we reviewed the bibliographies of the relevant articles identified through the searches for any additional articles that met the inclusion criteria.

## Eligibility criteria

Two reviewers (BP and SL) independently applied eligibility criteria to each of the potentially relevant articles. Eligible trials met the following inclusion criteria: (1) extracorporeal shockwave therapy was used as primary treatment; (2) patients were treated for nonunions, delayed unions or acute fractures; (3) articles were peer-reviewed; and (4) articles were written in English.

The reviewers obtained consensus on all inclusion status. Discrepancies were resolved by discussion or input of a third reviewer (SS).

### Assessment of study quality

Each article was read in full by two authors (BP and SL), who confirmed the inclusion criteria. Methodological criteria included randomization or presence of a comparison group in the case of observational studies, blinding (including surgeons, patients, outcomes assessors, and data analysts), the rate of loss to follow-up, and whether a sample size calculation was done and conducted a priori. These data were used to determine study quality using the criteria put forward by the GRADE working group.^25^ Using this approach, studies are classified into high, moderate, low or very low quality of evidence.^25^

### Data abstraction

Data were abstracted from each eligible study: (1) study characteristics including the sample size and the mean follow-up time; (2) patient characteristics including mean age, age range, and number of females and males; and (3) percentage of bone union after treatment.

## RESULTS

### Search results

**Figure 1:**
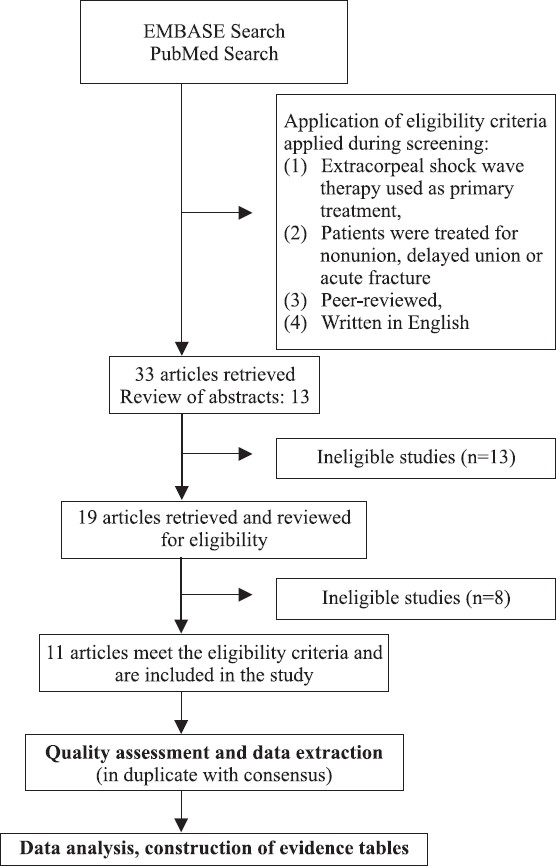
Flow chart of study process

Thirty-three potentially relevant studies were identified [Figure 1]. After reviewing the abstracts of these 33 studies, 22 of these articles were deemed ineligible: 6 articles were published in the non-English language, 1 article was a case report, 8 citations were reviews or non-peer-reviewed book chapters, 2 articles included patients reported on in a later series, 6 articles included soft tissue musculoskeletal diagnoses. Full-text versions were retrieved where possible and 11 articles met the inclusion criteria, and are included in this review.

### Nonunions and delayed unions

#### Methodological quality

One randomized controlled trial was identified in abstract form from the Proceedings of the American Academy of Orthopaedic Surgeons.^24^ This trial was randomized and blinded; however, further methodological criteria were not provided by the authors. Methodologically, all nine other clinical studies were single-center case series (low or very low grade of evidence using the GRADE working group classification) following a group of patients who were treated with shockwave therapy for a nonunion^8^,^9^,^20^,^22^,^25^–^28^ [Table 1]. Two case series assessed a subgroup of delayed unions to nonunions^8^,^26^ [Table 1]. One study blinded radiographic outcome assessors.^21^ No study reported an a priori sample size calculation. Two studies were prospective and seven were retrospective [Table 1].

**Table 1:** Study characteristics and outcomes for included studies assessing extracoporeal shockwave therapy on nonunion or delayed union

Author/Year	Study Design	N Patients	Mean Age	Follow-up	Diagnosis N(%)	Bone	ESWT	Union rate N (%)
Logan *et al*./2007	RCT	14	NA	3 yrs	Delayed Union	Long bones	3000 impulses EFD or kV not mentioned	No difference
Xu, *et al*./2008	Retrospective case series	69	38	96% Up to 90 months	Atrophic NU: 11 (15.9%) Hypertrophic NU: 58(84.1%)	Femur 22 Tibia 28 Humerus 1 3 Forearm 6	Femur/tibia: 6000 – 10000 impulses, 28kV anc EFD 0.62mJ/mm^2^	Overall: 50 (75.8%) AtrophicNU: 0(0%) Hypertrophic NU: 50 (91%)
							Humerus/Forearm 3000-4000 impulses, 24kV anc EFD 0.56 mJ/mm^2^	
Bara, *et al*./2007	Retrospective case series	81	Age range 12-89	6mths	Delayed union or NU	Tibia (49) Femur (13) Forearm (10) Humerus (5) Other (14)	1500–3000 impulse 20kv, wave pressui 500 bars within 1 microsecond Forearm: 1500 impulses All other bones: 3000 impulses	Overall: 67/81 (83%) 40/42 healed with DU (95%) 27/39 healed with NU (67%)
Biederman, *et al*./2003	Retrospective case series	73	42	96% 17mths	NU: 57 (78%) Delayed union: 16(22%) Hypertrophic NU: 34(61%) Atrophic NU: 22 (39%)	Long 2bone: 83%	Average 2900 impulses and 23kV	Overall: 32 (56%) AtrophicNU: 11 (50%) Hypertrophic NU: 21 (62%) Delayed union: 11 (93%)
Wang *et al*./2001	Prospective case series	72	39.4	76% 12mths	Hypertrophic NU: 38(52.8%) Atrophic NU: 13 (18%) NU with defect: 21 (29.2%)	Femur: 41 Tibia: 18 Humers: 7 Forearm:	2000-6000 impulses, 28kV	Overall: 44(80%) Atropic NU: 10(75%) Hypertrophic NU: 25 (80.6%) NU with defect: 6 (81.3%)
Schaden *et al*./2001	Retrospective case series	115	47.1	100% Up to 4 years	NU: 80(70%) Delayed union: 35 (30%)	Feumr: 12 Tibia: 34 Humerus: 5 Forearm: 14 Other: 50	1000–12000 impulses, 28kv Tibia/femur 0.4mJ/mm^2^ Scaphoid: 0.25–0.35mJ/ mm^2^	Overall: 87(75.7%) NU: 61 (76.3%) Delayed union: 26 (74.3%)
Rompe *et al*./2001 (possibly extension of patients from Vogel *et al*., 1997	Prospective case series	43	39.5	100: 9mths	Nonuion	Femur: 24 Tibia: 19	3000 impulses 0.6mJ/mm^2^	Overall: 31 (72%)
Vogel *et al*./1996	Retrospective case series	48	38	12mths	Nonuion	Femur: 17 Tibia: 19 Other lower extremity: 11	3000 impulses 0.6mJ/mm2	Overall: 29(60.4%)
Schleberger *et al*./1992	Retrospective case series	4	2 adults 2 pediatrk	NA	Nonunion	Humerus Tibia Metatarsal Tibia/fibula fusion	2000 impulses, 18kV	Overall: 3(75%)
Valchanou *et al*./1991	Retrospective case series	79	28	NA	Nonunion	Femur: 6 Tibia: 10 Humerus: 5 Forearm: 32 Other: 25	1000–4000 impulses, 1000-1700 bar	Overall: 70(85.4%)

ESWT: extracorporeal Shockwave therapy, NU: nonunion, EFD: energy flux density, kV: kilovolt

The average initial sample size across the clinical studies included in this review was 67 patients. Follow-up rates were fairly good among the included articles with reported ranges from 76 to 100%.^8^,^9^,^20^,^22^ The studies with the highest follow-up rate were of Logan *et al*. and Rompe *et al*. who did not report any patient lost to the follow-up.^21^ The article with the lowest follow-up rate was of Wang *et al*. with only 55 fractures available for radiographic data collection at 1 year.^9^

#### Patient characteristics

Patient information was not available from the one randomized trial identified; however, 14 patients were included in the trial. For the observational trials, the mean age varied for each study; however across the studies, the average mean age was approximately 39 years. All of the articles reported higher male than female ratios. The lowest female-to-male ratio was reported by Wang *et al*., with only 26% of the population being female.^9^ The closest-to-even ratios were reported by Biedermann *et al*. and Rompe *et al*. who both had 47% of their sample size as females.^8^,^21^

There was a medium range of follow-up periods for the articles included in this review. The studies varied from a mean follow-up time of 6 months up to ∼7.5 years.^8^,^9^,^20^,^22^,^27^,^28^ [Table 1].

#### Preoperative diagnoses

The preoperative diagnoses for the patients in the observational studies were either delayed healing or nonunions [Table 1]. Three of the articles went on further to describe the nonunions as hypertrophic or atrophic.^8^,^9^,^22^ In general, for the included trials, nonunion was defined as persistent fracture line with or without pain at the site for more than 6 months postfracture or no progression of healing on radiographs taken 3 months apart; delayed union was generally defined as delayed healing in less than 6 months postfracture. Four trials included only those patients with a nonunion^21^,^25^,^27^,^28^ while the remaining five included both nonunions and delayed unions.^8^,^9^,^20^,^22^,^29^ However, even given these criteria, patients both within studies and between studies were heterogeneous, having a number of different nonunion diagnoses (fracture versus osteotomy for example), as well as a number of different treatment modalities (nonoperative versus operative fixation often with intramedullary nailing or plate osteosynthesis) and differing lengths of time between fracture and the use of shockwave therapy.

#### Technical considerations

Depending on the bone, a different amount of intensity is applied. Less intensity is used for the smaller bones of the upper extremity as compared to a higher intensity for the lower extremity. Available data suggest that impulses of 1,500-3,000 were used for the upper extremity and impulses of 6,000 up to 12,000 were used for nonunions of the femur or tibia with differing kVs. As different machines may have different energies emitted per pulse, more standardized methods of calculating the energy per shockwave on tissue have been developed.^18^,^30^,^31^ These include the energy flux density, total energy flux, and peak pressure to name a few.^18^ The energy flux density is the amount of energy in a given amount of tissue (usually 1 mm^2^) at a given point in time and is a more standard measure of shockwave energy. Indeed, it has been suggested that measuring energy by laser hydrophones and reporting this as the energy flux density allows for more accurate reporting of the biologically available energy and thus aids comparing results from study to study.^16^,^31^ We attempt to report standardized energy values when available [Table 1].

In all trials where reported, the procedure was done in the operating room with C-arm localization. The fluoroscopic unit was, in some devices, incorporated into the shockwave machine. Most patients where reported required either general or regional anesthesia for treatment with extracorporeal shockwaves. We identified one disclosure statement stating that “no benefits in any form have been received or will be received from a commercial party related directly or indirectly to the subject of this article.”^9^

#### Union rate

Apart from the one RCT in abstract form, no significant difference in the union rate at 6 months was seen with the use of extracorporeal shockwaves as compared to controls. From the observational studies, the overall pooled result for bony unions using shockwave therapy on nonunions or delayed unions using a worst case scenario as regards follow-up is 471 united fractures out of 653, that is, a union rate of 72% (95% CI 69–76%]. Subgroup analysis of atrophic nonunions and hypertrophic nonunions revealed a statistically significant difference in union rates [Table 2]^9^,^22^. The union rate from available data for atrophic nonunion was 42% (95% CI 23–61%) and for hypertrophic nonunion was 78% (95% CI 70–86%) (*P* < 0.05). Indeed, Wang *et al*. reported a 0% union rate for patients with atrophic nonunions.^9^ The time until a full union was not clear in many studies. However, the average follow-up time was 1.8 years in which 72% of nonunion patients and 80% of delayed healing patients had already achieved unions. Trends were again observed that suggest that a union can be seen 3–6 months after the use of extracorporeal shockwave therapy.^14^,^20^,^22^

**Table 2:** Sub-group analysis of union rates between atrophic and hypertrophic nonunion, compiled from available data.

Union	Pooled estimate of union rate
Overall	72% [95%CI 69% – 76%]
Atrophic nonunion	42% [95% Cl 23% – 61%]
Hypertrophic nonunion	78% [95% Cl 70% – 86%]

#### Adverse events

No significant adverse events were reported in any of the trials. Rompe *et al*. suggest that transient local hematoma formation occurred; however, no other effects were reported and no effects on implants were identified.^21^ Other identified side effects included dermal erosion and transient local edema as well as petechial hemorrhages.^20^,^25^

### Acute fractures

#### Methodologic quality

One randomized trial was identified that assessed the effects of extracorporeal shockwaves on acute high-energy fractures [Table 3].^32^ This trial included only those fractures of the femur and tibia which required open reduction and internal fixation and had a fracture gap of <5 mm. The patients were pseudorandomized with the study group having a surgery on odd-numbered days of the week and the control group having a surgery on even-numbered days of the week. Follow-up was until 12 months with interim assessments at 1, 3, and 6 months. A blinded independent examiner performed the follow-up exams and a blinded radiologist was used for radiographic assessment. Two patients were excluded from the analysis, one from each group for deep infection and osteomyelitis. There were no crossovers.

**Table 3:** Study characteristics for trial involving acute fractures with nonunion event rate given

Author/ Year	Study Design	N Patients	Mean Age	Follow-up	Diagnosis N(%)	Bone	ESWT	Nonunion event rate N(%)
Wang *et al*., 2007	RCT Pseudorandomization Odd and even days	ECSW: 28 Control: 31	ECSW: 35.5 Control: 35.4	ECSW: 27/28 (96%) Control: 30/31 (97%)	High energy fracture with bone gap <5mm and non-articular	Femur Tibia	6000 impulses at 28kV	ECSW: 3/27 (11%)	
								Control: 6/30 (20%)

ESWT: extracorporeal Shockwave therapy, ECSW: extracorporeal Shockwave, RCT: randomized controlled trial

#### Results

Fifty-nine patients were enrolled with 28 in the study group and 31 in the control group. Prognostic factors such as open or closed fracture, nailing versus plate fixation, and mechanism of the injury were evenly distributed between groups. At all time points, the study population had a better pain score and weight-bearing status than the control group (*P*<0.01). Final follow-up (12 months) revealed a nonunion event rate of 11% (3 of 27) in the study group versus 20% (6 of 30) in the control group, and this was deemed statistically significant (*P*<0.001). All of the nonunions occurred in association with femoral fractures. This translates to a relative risk for nonunions using extracorporeal shockwave therapy of 0.54 (95% CI 0.152.01) or said another way, a 46% reduction in the risk of nonunions was seen when acute high-energy fractures with a fracture gap of <5 mm were treated with extracorporeal shockwave therapy as an adjuvant to open reduction and internal fixation. We have used review manager 5 (Cochrane Collaboration) software to create a Forest plot of these data [Table 4].^33^ Using this technique, we did not find a statistical difference in the event rate from the data provided but identified a trend for improved union rates with the use of extracorporeal shockwaves. It is possible that this discrepancy has arisen from the use of differing statistical tests or from the differential use of a one- or two-tailed test.

**Table 4:** Forrest plot of the relative risk of nonunion comparing extracorporeal shockwave and control group in acute high energy fractures, data from Wang *et al*., 2008

Study or subgroup	Extracorporeal shockwave events	Control				Risk ratio M-H, Fixed, 95% CI	Risk ratio M-H, Fixed, 95% CI
							
		Total	Events	Total	Weight		
Wang *et al*., 2008	3	27	6	30	100.0%	0.56 [0.15, 2.01]	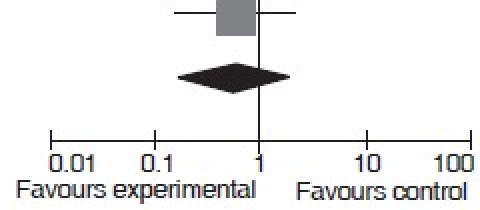
Total (95% CI)		27		30	100.0%	0.55 [0.15, 2.01]	
Total events	3		6				
Haterogeneity: Not applicable							
Test for overall effect: Z = 0.90 (*P* = 0.37)							

No adverse events were attributable to the shockwave device. Two patients developed deep infection, one in each group.

## DISCUSSION

Our review suggests that (1) current evidence from one RCT in abstract form suggests no effect of the use of extracorporeal shockwave therapy on union rate as compared to controls for delayed unions; however, this trial is limited by a very small sample size and the possibility of beta error. (2) Observational studies suggest there may be a beneficial effect of extracorporeal shockwave therapy on the healing of nonunions and delayed unions; however, these studies are limited by hetergeneous patient populations and low methodologic quality. (3) Current evidence from one pseudorandomized trial with methodological limitations suggests that there may be a trend toward decreased nonunions with the use of extracoporeal shockwave therapy (RR 0.56, 95% CI 0.152.01). (4) Current evidence from observational studies is insufficient to guide surgeons as to which patients may benefit from treatment; however, there are trends to suggest that atrophic nonunions may not respond significantly to extracorporeal shockwave therapy. (5) Given the significant methodological limitations and heterogeneity of patient populations seen with the current best evidence, conclusions should be seen in the light of hypothesis generating for the conduct of a large-scale randomized trial and interpreted clinically with caution.

Extracorporeal shockwave therapy has increasingly been used in fracture management and specifically, in its role as a nonoperative treatment strategy for nonunions or delayed unions. A potential downside however of this treatment modality as compared to other adjuvant bone-healing modalities such as low-intensity pulsed ultrasound and electrical stimulation is the necessity to undergo some sort of anesthesia.^34^ Indeed, shockwaves generate direct mechanical forces and subsequent cavitation forces with some microfracturing occurring potentially.^18^ This is inherently painful, and all studies that reported it used some form of general or regional anesthesia which has its own inherent risks. However, with a shockwave generation of <2,000 impulses, anesthesia may not be necessary.^34^ Secondly, X-ray localization is usually necessary which also incurs another dose of radiation.

Other potential issues include the differing mechanisms and machines to generate shockwaves. Indeed, these can be generated through electrohydraulic, piezoelectric, and electromagnetic systems, not to mention different manufacturers.^18^,^34^ Some authors suggest that there is no consensus as regards what constitutes high-, medium-, or low-energy impulses or for that matter if there is a correlation between dose and effect clinically.^18^,^34^ Thus, it is difficult to compare or assess intermachine variation.

### Strengths and limitations

Our review is strengthened by its comprehensive review and duplicate assessment of study quality. However, data pooling was based on low-quality evidence which lacked a control group as well as the bias-reducing measures of randomization, blinding, and in some instances a proscribed follow-up as well as the retrospective nature of some of the included studies. Indeed it has been shown that observational nonrandomized trials may over or underestimate the true treatment effect.^35^,^36^ The heterogeneity of the patient population with respect to location of the nonunions, previous surgical procedures, and duration of nonunions also limits the inferences and generalizability of these results.

### Evidence-based bottom line

Arguably the evidence in favor of shockwave use in nonunions, delayed unions or high-energy fractures as regards increased union rates would be a C recommendation.

Further research in this area in the form of a large-scale randomized trial is necessary to better answer the question of the effectiveness of extracorporeal shockwave therapy in union rates for both nonunions and acute fractures.
